# Age differences in psychological distress during the COVID-19 pandemic: March 2020 – June 2021

**DOI:** 10.3389/fpsyg.2023.1101353

**Published:** 2023-02-06

**Authors:** Ryan Best, JoNell Strough, Wändi Bruine de Bruin

**Affiliations:** ^1^Department of Psychology, West Virginia University, Morgantown, WV, United States; ^2^Price School of Public Policy, University of Southern California, Los Angeles, CA, United States; ^3^Dornsife Department of Psychology, University of Southern California, Los Angeles, CA, United States

**Keywords:** socioemotional selectivity theory, strength and vulnerability integration model, anxiety, depression, COVID-19 pandemic

## Abstract

In March 2020, COVID-19 brought illness, lockdowns, and economic turmoil worldwide. Studies from March–April 2020 reported increased psychological distress, especially among younger (vs. older) adults. Here, we examine whether age differences persisted in a 29-wave longitudinal survey conducted with an American national life-span sample over the first 16 months of the pandemic. Socio-emotional selectivity theory (SST) predicts that older age will be consistently associated with lower psychological distress due to life-span changes in motivation, while the strength and vulnerability integration model (SAVI) posits that age differences in psychological distress will diminish under prolonged stress. We find that younger adults consistently reported more psychological distress than older adults, though age differences did decrease over time. Prior diagnosis with anxiety or depression additionally predicted greater psychological distress throughout the study, but did not moderate age differences. We discuss implications for psychological theories of aging and interventions to reduce psychological distress.

## 1. Introduction

The World Health Organization posits that mental health is an integral part of the general health and well-being of people around the world (World Health Organization, 2022). Unfortunately, psychological distress became more common during the COVID-19 pandemic as compared to before, according to a recent meta-analysis across 61 studies covering Europe, North America and China (Robinson et al., 2022). The pandemic posed health and financial stressors for people of all ages (Kämpfen et al., 2020).

At the start of the pandemic, concerns were raised about stress-related mental health disorders in older adults because older adults experienced more severe complications, higher mortality, and more disruptions from COVID-19 (Vahia et al., 2020). However, initial studies suggested that older adults were actually less likely than younger adults to report psychological distress in March 2020 (Vahia et al., 2020; Bruine de Bruin, 2021). Older Americans also reported less negative and more positive emotions during the COVID-19 surge in April–May 2020 (Carstensen et al., 2020; Birditt et al., 2021). Similarly, older adult age was negatively correlated with self-reported anxiety and depression during the first months of the COVID-19 pandemic in Ireland (Hyland et al., 2021), Canada (Nwachukwu et al., 2020), and Spain (García-Fernández et al., 2020).

Long before the pandemic, it had also been demonstrated that older adults tend to have better emotional well-being than younger adults (Charles and Carstensen, 2010; Stone et al., 2010; Carstensen et al., 2011). The correlation between older adult age and reporting less psychological distress held after accounting for age differences in pre-pandemic mental health (Bruine de Bruin, 2021; Hyland et al., 2021). The finding that older adults experience less psychological distress than younger adults has long been termed ‘the well-being paradox’ because older adults report better emotional well-being despite being more likely to experience health problems, physical limitations, and loss of loved ones (Baltes and Baltes, 1990). The COVID-19 pandemic presented the opportunity to assess age differences in psychological distress in light of a serious and prolonged real-life stressor.

As the pandemic persisted for more than a year, two main theories of age differences in emotional well-being posit conflicting expectations for how older adults will fare emotionally. First, socioemotional selectivity theory (SST) posits that older adults will continue to report better emotional well-being despite persistent stressors, as compared to younger adults (Carstensen et al., 1999). Older adult age brings increasing awareness of the brevity and fragility of life, which motivates people to maximize emotional meaning in the ‘here and now’ (Carstensen, 2006). Inducing such a limited future time perspective has been associated with improved memory for positive relative to negative information, choosing to spend time with loved ones, pursuing attractive opportunities, and abandoning failing projects (Fung and Carstensen, 2006; Strough et al., 2014; Barber et al., 2016; Strough et al., 2019). From the perspective of SST, benefits of aging for emotional well-being are robust because they reflect life-span developmental changes in motivation (Reed et al., 2014).

In contrast, the strength and vulnerability integration model (SAVI) posits that older adults will only fare better than younger adults when it is relatively easy for them to downregulate their arousal (Charles, 2010). According to the SAVI model, age-related physiological vulnerabilities prevent older adults from downregulating arousal when stressors are sustained, serious, or unavoidable (Charles, 2010). Indeed, older adults reported less negative emotions than younger adults when experiencing a single stressor but more negative emotions when faced with stressors affecting multiple life domains (Wrzus et al., 2013). Older adults also reported less negative emotions than younger adults when stressful social relationships were avoidable, but this advantage disappeared when stressful social relationships were unavoidable (Birditt, 2014).

A few cross-sectional studies conducted during the COVID-19 pandemic show support for the SAVI model. In March and April 2020, American and Canadian older adults reported fewer daily stressors than younger adults, but there were no age differences in COVID 19-related stressors (Klaiber et al., 2021). These older adults did report fewer negative emotions than younger adults (Klaiber et al., 2021), which may not necessarily be a contradiction to SAVI. Another study conducted in the Netherlands in April–May 2020 found that age differences in negative emotions persisted during the pandemic but were smaller as compared to the presumably less stressful period before the pandemic (Sun and Sauter, 2021).

Remarkably few longitudinal studies have examined whether age differences in psychological distress held over time during the ongoing COVID-19 pandemic. Those longitudinal studies did not test whether age differences in mental health reflected the SST or SAVI models over time, but rather analyzed age differences in changes in psychological distress compared to pre-pandemic levels. For example, a longitudinal study conducted in the US found that participants in four age groups (18–34, 35–49–50-64, 65+) showed similar increases in psychological distress in March–April 2020 compared to pre-pandemic levels, but their psychological distress returned to pre-pandemic levels by July 2020 (Daly and Robinson, 2021a). A longitudinal study conducted in the United Kingdom found that mental health problems initially increased the most among younger adults (18–34 years) and the least among middle-aged (50–64 years) and older adults (65+ years) as compared to pre-pandemic levels, but age differences were reduced by June 2020 due to larger declines in younger (vs. older) age groups (Daly et al., 2020). Subsequent studies from the United Kingdom found that psychological distress decreased to pre-pandemic levels for all age groups by September, 2020 (Daly and Robinson, 2021b), though a subsequent wave of COVID-19 infections brought increases in psychological distress from September 2020 to January 2021 (Daly and Robinson, 2022). Indeed, deaths attributed to COVID-19 showed peaks at the beginning of the pandemic, as well as in the fall and winter of 2020–2021 and 2021–2022 (Centers for Disease Control and Prevention, 2022). These findings underscore the importance of continuing to track age differences in psychological distress as the COVID-19 pandemic persists, and produces different waves.

This study examines age differences in psychological distress over the course of the pandemic. We conducted a secondary analysis of longitudinal data from an adult life-span sample responding to surveys spanning the first 16 months of the ongoing COVID-19 pandemic, from March 2020 through June 2021. Using mixed models, we investigated changes in age differences in psychological distress across the first 16 months of the COVID-19 pandemic by testing the interaction between age and survey wave. Our research question asked: Do age differences in the likelihood of psychological distress vary across the first 16 months of the COVID-19 pandemic even after accounting for age differences in pre-pandemic anxiety and depression? Our hypothesis, based on expectations from SST, was that the association between older age and a lower likelihood of psychological distress would remain stable throughout the entire first 16 months of the pandemic. Our alternative hypothesis, based on expectations from the SAVI model, was that the association between older age and a lower likelihood of psychological distress would decrease across the 16 months. Additionally, we examined whether age differences in psychological distress were more pronounced among individuals who had previously been diagnosed with anxiety or depression, which may have made them more vulnerable to psychological distress during the pandemic (Bruine de Bruin, 2021).

## 2. Methods

### 2.1. Transparency and openness

Our secondary data analyses use data from the University of Southern California’s Understanding America Study (UAS). De-identified data and codebooks for all surveys are publicly available.^1^ Data exclusions and measures relevant to our analyses are described in the following sections. Mixed-model analyses were conducted using the lme4 (Bates et al., 2015) and lmerTest (Kuznetsova et al., 2017) packages in R (R Core Team, 2020) using the RStudio interface (RStudio Team, 2020). The analytic code needed to reproduce the analyses is included in the online supplementary materials. The study design, hypotheses, and analytic plan were not pre-registered.

### 2.2. Participants

Participants were members of the UAS, which is a nationally representative probability-based sample (*N* ⁓ 9,500). Individuals without Internet access and/or an internet accessible device received tablets, Internet access, or both. Panel members provide consent to participate in UAS surveys, and for anonymized survey data to “be used in future research studies or shared with other researchers.” All surveys were approved by the USC human subjects committee internal review board. A subset of 8,628 UAS participants aged 18–101 (*Mean* = 49.22, *SD* = 16.35) completed at least one of twenty-nine survey waves, described in more detail below. This sample size is sufficient for detecting effect sizes of *r* < 0.03 (given *p* < 0.05 and *B* = 0.80).

### 2.3. Survey waves

Participants were prompted to respond to 29 survey waves spanning 16 months between March 10, 2020 and June 30, 2021 (see Supplemental Table S1 for survey wave schedule). The initial survey was distributed in the field from March 10–31, 2020. Between April 1, 2020 and March 16, 2021, survey waves occurred every 2 weeks, with 1/14 of participants being invited to complete the survey each day. From March 17, 2021 to July 20, 2021, after the first year of data collection, survey frequency was reduced to monthly surveys because of a reduction in funding. Analyses of changes in psychological distress across the first eight survey waves (until June 2020) were previously reported elsewhere (Daly and Robinson, 2021a). Additional information about survey methodology is provided by Kapteyn et al. (2020) and on the UAS website.^2^

On average, participants completed more than two-thirds of survey waves (*M*_waves_ = 20.82, *SD* = 9.65, Median_waves_ = 26), with 3,007 completing all waves and 5,608 completing 20 or more. Separate mixed-effects logistic regression models were fit to the data to predict the likelihood of completing ≥20 survey waves or all 29 survey waves, including participant as a random effect and age, gender, marital status, race, education, income, psychological distress, and diagnosis of depression or anxiety prior to the COVID-19 pandemic as fixed factors (see Supplemental Table S2). In both models, the only significant predictor of survey wave completion was age, where increased age was associated with an increased likelihood of completing more survey waves. Demographic factors, aside from age, were generally not predictive of survey completion. Further analyses were conducted on the full sample (*N* = 8,628), removing cases listwise if missing data were encountered.

#### 2.3.1. Psychological distress

At each survey wave, participants completed the validated 4-item Patient Health Questionnaire (PHQ-4), which is a diagnostic tool that assesses whether or not individuals show signs of anxiety (“feeling nervous, anxious, or on edge”) and depression (“feeling, down, depressed or hopeless”) over the past 2 weeks (Kroenke et al., 2009; Löwe et al., 2010). Response options included not at all (0), several days (1), more than half the days (2), and nearly every day (3). Internal consistency was sufficient to warrant summation of scores for the overall scale (*α* > 0.92 across survey waves). Following the convention to use the dichotomized score as a diagnostic tool (Löwe et al., 2010), we treated scores of ≥6 as reflecting signs of depression and anxiety (=1) and scores <6 as not (=0).

#### 2.3.2. Previous diagnosis

In survey waves 4–29, participants were asked if they had ever been diagnosed with anxiety or depression by a medical professional. A follow-up question asked if this diagnosis was received prior to or after March 10th, 2020. These questions were added to suvery waves 4-29 because analyses conducted on initial waves showed that pre-pandemic diagnosis with anxiety and depression was a strong predictor of psychological distress during the pandemic, but information about pre-pandemic diagnosis was reported in 2019 by only 85% of participants (e.g., Bruine de Bruin, 2021). We used these items to calculate a binary variable differentiating between individuals who had received a diagnosis of anxiety or depression prior to March 10th, 2020 (coded as 1) and those who had not received a diagnosis of anxiety and/or depression prior to March 10th, 2020 (coded as 0). Of the 8,283 participants who responded to this item, 2,154 (26.0%) reported a diagnosis of anxiety or depression before the onset of the COVID-19 pandemic.

#### 2.3.3. Demographics

The UAS collects participants’ demographic information every 3 months. Demographic variables included in the reported analysis are gender (1 = Male, 0 = Female), race (1 = White/Caucasian, 0 = All other responses), marital status (1 = Married, 0 = Not married), annual household income (1 = Greater than or equal to $75,000, 0 = Less than $75,000) and education (1 = Bachelor’s degree or higher, 0 = no college degree). These variables were chosen for inclusion in the statistical models to control for demographic variance in psychological distress (Hossain et al., 2020; Bruine de Bruin, 2021).

### 2.4. Analyses

To investigate age differences in psychological distress throughout the COVID-19 pandemic, the data were fit to a logistic mixed-model predicting psychological distress, as indicated by PHQ-4 scores of 6 or greater, including participant as a random factor with age, measurement wave, and previous anxiety or depression diagnosis as fixed factors. Using a hierarchical procedure, subsequent models added interaction terms. Specifically, the second step added two-way interactions between age and survey wave, and between age and pre-pandemic anxiety or depression diagnosis. Last, we included the three-way interaction term between age, pre-pandemic anxiety or depression diagnosis, and survey wave. Control variables were entered as fixed factors, including dichotomous variables for male vs. female gender, white vs. minority race/ethnicity, married vs. unmarried status, annual income ≥$75,000 vs. less, and college education vs. not. Conclusions were unaffected by whether or not these covariates were included. Reported analyses (see Table 1; Supplemental Table S3 for standard errors and z values) were weighted using values provided by the UAS for each survey to make the data socio-demographically representative of the US adult population (see Angrisani et al., 2019; see Supplemental Tables S4,S5 for unweighted analyses). A correlation matrix for all model variables can be found in Supplemental Table S6.

**Table 1:** Logistic mixed-models predicting PHQ-4 indicator of psychological distress (scores ≥6) – including 95% CIs of estimates.

	Model 1			Model 2			Model 3		
Predictor	*b*	95% CI of *b*	*p*	*b*	95% CI of *b*	*p*	*b*	95% CI of *b*	*p*
Intercept	−1.6	[−1.88, −1.33]	<0.001	−1.63	[−1.97, −1.28]	<0.001	−1.6	[−1.96, −1.25]	<0.001
Age	−0.03	[−0.04, −0.03]	<0.001	−0.03	[−0.04, −0.02]	<0.001	−0.03	[−0.04, −0.02]	<0.001
Wave	−0.02	[−0.02, −0.02]	<0.001	−0.01	[−0.02, <−0.01]	0.03	−0.01	[−0.02, <−0.01]	0.04
Prior diagnosis	1.96	[1.80, 2.12]	<0.001	1.32	[0.78, 1.86]	<0.001	1.25	[0.63, 1.86]	<0.001
Age * Wave				<−0.01	[<−0.01, <−0.01]	<0.001	<−0.01	[<−0.01, <−0.01]	0.01
Age * Diagnosis				0.01	[<−0.01, 0.02]	0.08	0.01	[<−0.01, <0.02]	0.07
Wave * Diagnosis				0.01	[0.01, 0.02]	<0.001	0.02	[<−0.01, <0.04]	0.04
Age * Wave * Diagnosis							<−0.01	[<−0.01, <0.01]	0.59
**Control variables**									
Male	−0.56	[−0.71, −0.40]	<0.001	−0.55	[−0.72, −0.39]	<0.001	−0.55	[−0.72, −0.39]	<0.001
White	<−0.01	[−0.18, 0.18]	0.97	<0.01	[−0.28, 0.19]	0.97	<0.01	[−0.18, 0.19]	0.97
Annual Income >$75 k	−0.29	[−0.42, −0.16]	<0.001	−0.29	[−0.42, −0.15]	<0.001	−0.29	[−0.42, −0.16]	<0.001
Married	−0.44	[−0.58, −0.30]	<0.001	−0.45	[−0.60, −0.30]	<0.001	−0.45	[−0.60, −0.30]	<0.001
Bachelor’s Degree	0.05	[−0.10, 0.20]	0.53	0.05	[−0.11, 0.21]	0.56	0.05	[−0.11, 0.21]	0.55

Psychological distress was measured with the Patient Health Questionnaire (PHQ-4), and scores ≥6 indicated anxiety and depression. Variable titled “Wave” refers to survey wave. Variable titled “Diagnosis” indicates a diagnosis of anxiety or depression prior to October 03, 2022. Addition of the two-way interaction terms significantly improved model fit, *χ*^2^ = 37.67, *p* < 0.001. Addition of the three-way interaction term did not improve model fit, *χ*^2^ = 1.43, *p* = 0.71.

## 3. Results

**Figure 1:**
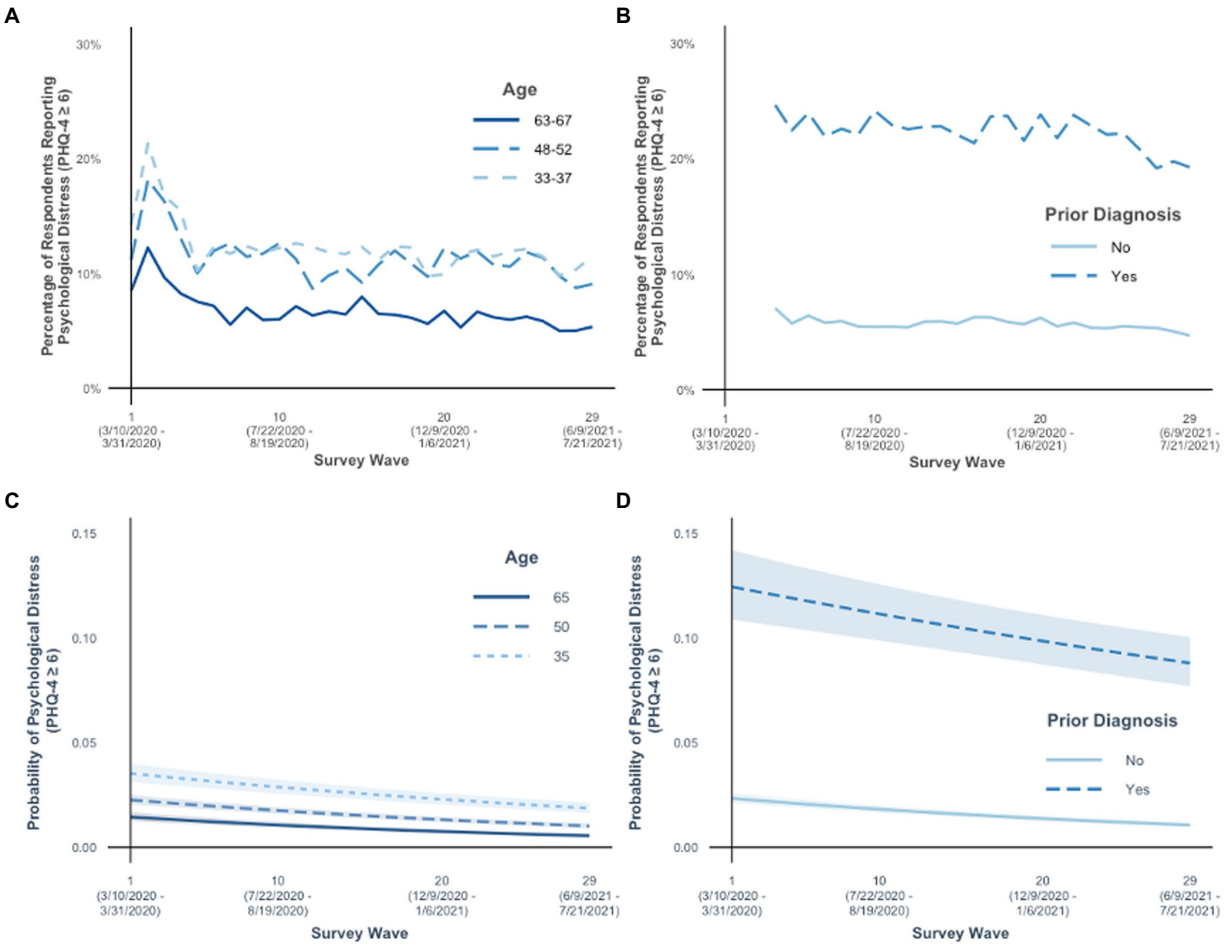
Psychological distress was measured with the Patient Health Questionnaire (PHQ-4), and scores [insert: greater than or equal to symbol]6 indicated anxiety and depression. Percent of people in the sample with psychological distress are reported for **(A)** age by survey wave and **(B)** prior diagnosis of anxiety and depression by survey wave. Predicted probabilities, conversions of the logit coefficients computed in the associated logistic regression models, are reported for the **(C)** age by survey wave interaction and **(D)** prior diagnosis of anxiety and depression by survey wave interaction. Shaded areas around lines in panels **(C)–(D)** indicate 95% confidence intervals. Age was included in statistical models as a continuous variable but separated into age groups for panels **(A)** and **(C)** to aid interpretation of the interaction of age and survey wave. In panel **(A)**, age groups are represented using 5-year age ranges representing approximately the sample mean (49.22 years) and one standard deviation above (65.57 years) and below (32.86 years) the mean age. In panel **(C)**, the interaction effect is demonstrated by showing the simple slopes for ages approximating the sample mean and one standard deviation above and below the mean age.

Older adult age was negatively associated with reporting psychological distress (Table 1, Model 1), but age differences decreased slightly across survey waves (Table 1, Model 2). These findings held while controlling for prior diagnosis with anxiety or depression. Figures 1A–B show descriptive statistics and Figures 1C–D show predicted probabilities associated with the logistic regression models. As seen in Figures 1A and 1C, at each survey wave, older adults were consistently less likely to report psychological distress than younger adults. Older adults’ likelihood of reporting psychological distress remained relatively stable throughout the pandemic, while it varied relatively more for younger adults. Age differences were largest at the beginning of the pandemic, when younger adults expressed the most psychological distress. Age differences were somewhat smaller as the pandemic progressed, due to younger adults’ reduced likelihood of reporting psychological distress across survey waves. The positive associations of age with psychological distress were smaller after accounting for prior diagnosis and demographics (Figures 1C vs. 1A).

Additionally, we found that individuals with (vs. without) a prior diagnosis of anxiety or depression were more likely to report psychological distress (Table 1, Model 1), but that difference decreased somewhat across survey waves (Table 1, Model 2). Prior diagnosis did not moderate reported changes in age differences across survey wave in psychological distress (Table 1, Model 3). At each survey wave, individuals with a prior diagnosis were consistently more likely to report psychological distress than individuals without a prior diagnosis (Figures 1B, D). Individuals with a prior diagnosis reported the greatest psychological distress at the start of the pandemic and throughout the survey waves, and then were slightly less likely to do so over time. Individuals without a prior diagnosis were relatively less likely to report psychological distress, and their likelihood of doing so remained relatively stable over time.

## 4. Discussion

At the start of the pandemic, older adults were less likely to report psychological distress than younger adults (Bruine de Bruin, 2021). SST predicts that these age differences will persist as the pandemic continues, because older adults are motivated to optimize their emotional experiences in the limited life that they have left (Carstensen et al., 1999). SAVI posits that benefits of aging for emotional well-being will be reduced or eliminated in the face of stressors that are sustained, serious and unavoidable – such as the COVID-19 pandemic (Charles, 2010). Our findings align with the SST model. We found that, over the first 16 months of the pandemic, older adults consistently were less likely to report psychological distress than younger adults. Although age differences decreased slightly over time, they did not follow SAVI’s prediction that older adults’ psychological distress would increase over time. Rather, younger adults showed less psychological distress over time after an initial peak at the start of the pandemic.

Our findings may appear to contradict two studies that did support SAVI. The first study found that in March–April 2020, American and Canadian older adults reported fewer daily stressors than younger adults, but there were no age differences in COVID 19-related stressors (Klaiber et al., 2021). However, when asked about their emotional well-being, these older adults did report less negative emotions than younger adults, in line with our study, suggesting that older adults were emotionally coping better than younger adults with the stressors they faced (Klaiber et al., 2021). The second study found that age differences in negative emotions were less pronounced at the start of the pandemic than before the pandemic among adults in the Netherlands in April–May 2020 (Sun and Sauter, 2021). Our study examined a different time period, and focused on trajectories of anxiety and depression in U.S. adults through the first 16 months of the pandemic. Over this time period, age differences diminished over time due to a decline in psychological distress in younger adults (see Figure 1A). Combined, these findings suggest that older adults generally fared better than younger adults at any time point before or during the pandemic – though the degree to which they fared better varied over time and, potentially, with the stressors they experienced.

Although our study had no measure of pandemic stressors, other studies suggest that younger adults may have experienced more pandemic-related life change, social isolation, and negative relationships than older adults did (Birditt et al., 2021). Among older adults, isolation and disruption to family relationships were also commonly reported pandemic stressors (Heid et al., 2021; Whitehead and Torossian, 2021). Older adults may have found it easier to cope with these stressors, perhaps by applying coping strategies learned from living through other major historical events (Lind et al., 2021). Specifically, older adults may have derived more support from their social network during the pandemic, with one study reporting that the association between age and increased positive emotional experiences could be partially explained by older adults reporting a greater closeness to friends (Cavallini et al., 2021). Furthermore, during the pandemic, older adults were more likely than younger adults to report using problem-focused and proactive coping strategies, and less likely to report counterproductive coping strategies such as ruminating about stressors (Dworakowski et al., 2021; Pearman et al., 2021; Young et al., 2021). Potentially, younger adults learned to apply these strategies as the COVID-19 pandemic went on, leading to the observed decrease in younger adults’ psychological distress following an initial peak.

Additional research is needed to learn more about the underlying mechanisms for the reported age differences in psychological distress, and their persistence over the course of the pandemic. Following from SST, younger adults may experience more distress than older adults when sociocultural events highlight the brevity of life because such events conflict with their future-oriented goals (Fung and Carstensen, 2006). Older adults appraised the pandemic as less disruptive to their goals (Young et al., 2021). In contrast, SAVI suggests that age-related strengths in downregulating arousal may have helped older adults to avoid or reduce the severity of pandemic-related stressors. However, SAVI also predicts that older adults find it harder to keep up this downregulation in the face of persistent stressors.

Having a prior diagnosis of anxiety or depression did not moderate the relationship between age and psychological distress over time. However, having such a prior diagnosis was a stronger predictor than age of having an increased likelihood of reporting psychological distress. The association between prior diagnosis and increased likelihood of psychological distress decreased somewhat across survey waves, but still, individuals with (vs. without) a prior diagnosis had an increased likelihood of distress across all survey waves. Interventions may be needed to help people of all ages to manage their psychological distress as the COVID-19 pandemic persists. Telemedicine was already established as a viable alternative to in-person care prior to the pandemic (García-Lizana and Muñoz-Mayorga, 2010). There is also evidence that self-administered computer-based cognitive behavioral therapy (Grist and Cavanagh, 2013) and smartphone apps (Firth et al., 2017) are effective tools for depression self-management. Delivering mindfulness-based cognitive therapy and mindfulness-based stress reduction through videoconferencing may also be feasible (Moulton-Perkins et al., 2022).

One limitation of this study is that individuals with mental or physical health problems may have been less likely to respond to the survey, potentially reducing the representativeness of the sample, especially of participating older adults. However, pre-pandemic diagnosis with anxiety or depression was not related to completion of survey waves for participants already taking part in the study. Another limitation of the current study is the focus on negative emotional experiences; positive emotions were not measured. Positive emotional experiences are a distinct construct and may have yielded different results. Yet, initial studies suggest that older adult age is associated with an increase in positive emotions over the course of the pandemic (Carstensen et al., 2020; Cavallini et al., 2021; but see also Ceccato et al., 2021).

Although our findings indicate persistent age differences in psychological distress across 16 months in a sample weighted to be representative of the US population and similar age differences have been reported for responses to natural disasters (Cherry et al., 2021), these findings may not generalize to future cohorts or other types of stressors. Individuals develop within historical contexts. Economic downturns and the rise of technology were associated with decreases in the emotional well-being of middle-aged adults in the 2010s compared to the 1990s such that age-related advantages for emotional well-being may be less evident for this cohort (Almeida et al., 2020). In addition, historical events have a greater impact when they occur earlier in adulthood (Stewart and Healy, 1989) and COVID-19 has increased economic and health disparities (Kämpfen et al., 2020). Thus, the current cohort of younger adults may show substantial heterogeneity in emotional well-being as they age.

Together, our findings suggest that older adults fared better emotionally than did younger adults during the first 16 months of the pandemic. Further research is necessary to investigate whether older age continues to protect against psychological distress as stressors related to climate change, inflation, supply-chain shortages, and the war in Ukraine accumulate and combine with those related to COVID-19. Knowledge of factors underlying age-related reductions in psychological distress can be leveraged to develop new interventions and refine therapies to reduce psychological distress in people of all ages (World Health Organization, 2022).

## Data availability statement

Publicly available datasets were analyzed in this study. This data can be found at: https://uasdata.usc.edu/page/Covid-19+Data.

## Ethics statement

The studies involving human participants were reviewed and approved by USC Human Subjects Committee Internal Review Board. The patients/participants provided their written informed consent to participate in this study.

## Author contributions

RB, JS, and WB conceived the study, interpreted the results, and drafted and edited the manuscript. RB conducted the analyses. All authors contributed to the article and approved the submitted version.

## Funding

Data collection was supported and conducted by USC's Center for Economic and Social Research. Author WBdB was supported by the National Science Foundation (#2028683) and USC's Schaeffer Center for Health Policy and Economics Schaeffer Center for Health Policy and Economics. The collection of the UAS COVID-19 survey data is supported in part by the Bill & Melinda Gates Foundation and by grant U01AG054580 from the National Institute on Aging.

## Conflict of interest

The authors declare that the research was conducted in the absence of any commercial or financial relationships that could be construed as a potential conflict of interest.

## Publisher’s note

All claims expressed in this article are solely those of the authors and do not necessarily represent those of their affiliated organizations, or those of the publisher, the editors and the reviewers. Any product that may be evaluated in this article, or claim that may be made by its manufacturer, is not guaranteed or endorsed by the publisher.

## Footnotes

^1^ https://uasdata.usc.edu/

^2^ https://uasdata.usc.edu/index.php
