# Supplementary material for: Age differences in psychological distress during the COVID-19 pandemic: March 2020 – June 2021
Source: Front Psychol. 2023 Feb 6;14:1101353. doi: 10.3389/fpsyg.2023.1101353 (PMC9939750; doi:10.3389/fpsyg.2023.1101353)
Supplement: Supplementary file 1 [file Table_1.DOCX]

Supplementary Material

**Supplemental Table 1**

*Survey waves and corresponding dates.*

| Survey Wave | Start Date | End Date |
| --- | --- | --- |
| 1 | 03/10/2020 | 03/31/2020 |
| 2 | 04/01/2020 | 04/28/2020 |
| 3 | 04/15/2020 | 05/13/2020 |
| 4 | 04/29/2020 | 05/26/2020 |
| 5 | 05/13/2020 | 06/09/2020 |
| 6 | 05/27/2020 | 06/23/2020 |
| 7 | 06/10/2020 | 07/08/2020 |
| 8 | 06/24/2020 | 07/22/2020 |
| 9 | 07/08/2020 | 08/05/2020 |
| 10 | 07/22/2020 | 08/19/2020 |
| 11 | 08/05/2020 | 09/02/2020 |
| 12 | 08/19/2020 | 09/16/2020 |
| 13 | 09/02/2020 | 09/30/2020 |
| 14 | 09/16/2020 | 10/14/2020 |
| 15 | 09/30/2020 | 10/27/2020 |
| 16 | 10/14/2020 | 11/11/2020 |
| 17 | 10/28/2020 | 11/25/2020 |
| 18 | 11/11/2020 | 12/09/2020 |
| 19 | 11/25/2020 | 12/23/2020 |
| 20 | 12/09/2020 | 01/06/2020 |
| 21 | 12/23/2020 | 01/20/2020 |
| 22 | 01/06/2021 | 02/03/2021 |
| 23 | 01/20/2021 | 02/17/2021 |
| 24 | 02/02/2021 | 03/03/2021 |
| 25 | 02/17/2021 | 03/30/2021 |
| 26 | 03/17/2021 | 04/27/2021 |
| 27 | 04/14/2021 | 05/25/2021 |
| 28 | 05/12/2021 | 06/22/2021 |
| 29 | 06/09/2021 | 07/21/2021 |

Note: From 04/01/2020 to 02/17/2021 (waves 2-25), surveys encompassed two weeks, with 1/14 of participants invited on each day. Participants were given up to two weeks to complete the questionnaire. As a result, the total time that each survey was in the field was approximately 4 weeks.

**Supplemental Table 2**

*Predictors of survey wave completion in the UAS Understanding Coronavirus in American Sample.*

|  | ≥ 20 waves | | | |  | All Waves | | | |
| --- | --- | --- | --- | --- | --- | --- | --- | --- | --- |
| Predictor | *b* | *SE* | *z* | *p* |  | *b* | *SE* | *z* | *p* |
| Intercept | -1.82 | 0.84 | -2.17 | .03 |  | -8.91 | 0.97 | -9.23 | <.001 |
| Age | 0.10 | 0.01 | 7.18 | <.001 |  | 0.12 | 0.02 | 7.92 | <.001 |
| Male | -0.25 | 0.46 | -0.54 | .59 |  | 0.31 | 0.51 | 0.61 | .55 |
| Income ≥ $75k | 0.37 | 0.43 | 0.86 | .39 |  | 0.25 | 0.46 | 0.54 | .59 |
| White | 0.05 | 0.53 | 0.09 | .93 |  | 0.41 | 0.61 | 0.68 | .49 |
| Married | 0.19 | 0.45 | 0.43 | .67 |  | 0.06 | 0.50 | 0.12 | .90 |
| College Degree | 0.59 | 0.47 | 1.26 | .21 |  | 0.93 | 0.52 | 1.79 | .07 |
| ^a^ Psychological distress | -0.03 | 0.05 | -0.69 | .49 |  | -0.02 | 0.06 | -0.42 | .67 |
| ^b^ Anxiety/Depression  Diagnosis | -0.76 | 0.52 | -1.47 | .14 |  | -.042 | 0.59 | -0.72 | .47 |

Note: The table presents two separate mixed-effects logistic regression models, predicting the likelihood of completing 20 or more survey waves in the left columns and likelihood of completing all 29 survey waves in the right column. ^a^Psychological distress was measured with the Patient Health Questionnaire (PHQ-4), and scores ≥ 6 indicated anxiety and depression. ^b^Anxiety/Depression Diagnosis refers to diagnoses prior to 03/10/2020.

**Supplementary Table 3**

*Logistic Mixed-models Predicting Psychological Distress*

|  | Model 1 | | | | Model 2 | | | | Model 3 | | | |
| --- | --- | --- | --- | --- | --- | --- | --- | --- | --- | --- | --- | --- |
| Predictor | *b* | *SE* | *z* | *p* | *b* | *SE* | *z* | *p* | *b* | *SE* | *z* | *p* |
| Intercept | -1.60 | 0.14 | -11.43 | <.001 | -1.63 | 0.17 | -9.64 | <.001 | -1.6 | 0.17 | -9.22 | <.001 |
| Age | -0.03 | <0.01 | -13.46 | <.001 | -0.03 | <0.01 | -9.41 | <.001 | -0.03 | <0.01 | -9.16 | <.001 |
| Wave | -0.02 | <0.01 | -13.57 | <.001 | -0.01 | <0.01 | -2.16 | .03 | -0.01 | 0.01 | -2.10 | .04 |
| Prior Diagnosis | 1.96 | 0.08 | 24.36 | <.001 | 1.32 | 0.25 | 5.19 | <.001 | 1.25 | 0.29 | 4.30 | <.001 |
| Age * Wave |  |  |  |  | <-0.01 | <0.01 | -3.71 | <.001 | <-0.01 | <0.01 | -2.67 | .01 |
| Age * Diagnosis |  |  |  |  | 0.01 | 0.01 | 1.78 | .08 | 0.01 | 0.01 | 1.80 | .07 |
| Wave * Diagnosis |  |  |  |  | 0.01 | <0.01 | 4.61 | <.001 | 0.02 | 0.01 | 2.02 | .04 |
| Age * Wave * Diagnosis |  |  |  |  |  |  |  |  | <-0.01 | <0.01 | -0.54 | .59 |
| *Control variables* |  |  |  |  |  |  |  |  |  |  |  |  |
| Male | -0.56 | 0.08 | -7.05 | <.001 | -0.55 | 0.08 | -7.00 | <.001 | -0.55 | 0.08 | -7.00 | <.001 |
| White | <-0.01 | 0.09 | -0.04 | .97 | <0.01 | 0.09 | 0.04 | .97 | <0.01 | 0.09 | 0.04 | .97 |
| Annual Income >$75k | -0.29 | 0.07 | -4.27 | <.001 | -0.29 | 0.07 | -4.21 | <.001 | -0.29 | 0.07 | -4.23 | <.001 |
| Married | -0.44 | 0.07 | -6.16 | <.001 | -0.45 | 0.07 | -6.34 | <.001 | -0.45 | 0.07 | -6.34 | <.001 |
| Bachelor’s Degree | 0.05 | 0.08 | 0.63 | .53 | 0.05 | 0.08 | 0.58 | .56 | 0.05 | 0.08 | 0.59 | .55 |

Note: Psychological distress was measured with the Patient Health Questionnaire (PHQ-4), and scores ≥ 6 indicated anxiety and depression. Variable titled “Wave” refers to survey wave. Variable titled “Diagnosis” indicates a diagnosis of anxiety or depression prior to 3/10/2022. Addition of the two-way interaction terms significantly improved model fit, χ^2^ = 37.67, *p* < .001. Addition of the three-way interaction term did not improve model fit, χ^2^ = 1.43, *p* = .71.

**Supplementary Table 4**

*Unweighted Logistic Mixed-models Predicting Psychological Distress– Including 95% CIs of Estimates*

|  | Model 1 | | | | Model 2 | | | | Model 3 | | | |
| --- | --- | --- | --- | --- | --- | --- | --- | --- | --- | --- | --- | --- |
| Predictor | *b* | 95% CI of *b* | | *p* | *b* | 95% CI of *b* | | *p* | *b* | 95% CI of *b* | | *p* |
| Intercept | -1.39 | [-1.68, -1.11] | | <.001 | -1.42 | [-1.77, -1.08] | | <.001 | -1.38 | [-1.73, -1.03] | | <.001 |
| Age | -0.04 | [-0.04, -0.03] | | <.001 | -0.04 | [-0.04, -0.03] | | <.001 | -0.04 | [-0.04, -0.03] | | <.001 |
| Wave | -0.02 | [-0.02, -0.02] | | <.001 | -0.01 | [-0.02, <0.01] | | 0.06 | -0.01 | [-0.02, <-0.01] | | .04 |
| Prior Diagnosis | 1.96 | [1.79, 2.13] | | <.001 | 1.40 | [0.86, 1.94] | | <.001 | 1.26 | [0.65, 1.88] | | <.001 |
| Age * Wave |  |  |  |  | <-.001 | [<-0.01, <-0.01] | | .002 | <-.001 | [<-0.01, <-0.01] | | .04 |
| Age * Diagnosis |  |  |  |  | 0.01 | [<0.01, 0.02] | | .11 | 0.01 | [<-0.01, 0.02] | | .06 |
| Wave * Diagnosis |  |  |  |  | 0.01 | [<0.01, 0.02] | | .003 | 0.02 | [<-0.01, 0.04] | | .07 |
| Age * Wave * Diagnosis |  |  |  |  |  |  |  |  | <-.001 | [<-0.01, <0.01] | | .35 |
| *Control variables* |  |  |  |  |  |  |  |  |  |  |  |  |
| Male | -0.46 | [-0.62, -0.29] | | <.001 | -0.46 | [-0.62, -0.29] | | <.001 | -0.46 | [-0.62, -0.29] | | <.001 |
| White | -0.08 | [-0.27, 0.10] | | .37 | -0.08 | [-0.26, 0.11] | | 0.40 | -0.08 | [-0.26, 0.11] | | .40 |
| Annual Income >$75k | -0.20 | [-0.34, -0.07] | | .002 | -0.20 | [-0.34, -0.07] | | .002 | -0.21 | [-0.34, -0.07] | | .002 |
| Married | -0.40 | [-0.55, -0.26] | | <.001 | -0.41 | [-0.56, -0.26] | | <.001 | -0.41 | [-0.56, -0.26] | | <.001 |
| Bachelor’s Degree | -0.13 | [-0.29, 0.03] | | .11 | -0.13 | [-0.29, 0.03] | | .11 | -0.13 | [-0.29, 0.03] | | .11 |

Note: Psychological distress was measured with the Patient Health Questionnaire (PHQ-4), and scores ≥ 6 indicated anxiety and depression. Variable titled “Wave” refers to survey wave. Variable titled “Diagnosis” indicates a diagnosis of anxiety or depression prior to 3/10/2022. Addition of the two-way interaction terms significantly improved model fit, χ^2^ = 26.23, *p* < .001. Addition of the three-way interaction term did not improve model fit, χ^2^ = 1.02, *p* = .31.

**Supplementary Table 5**

*Unweighted Logistic Mixed-models Predicting Psychological Distress*

|  | Model 1 | | | | Model 2 | | | | Model 3 | | | |
| --- | --- | --- | --- | --- | --- | --- | --- | --- | --- | --- | --- | --- |
| Predictor | *b* | *SE* | *z* | *p* | *b* | *SE* | *z* | *p* | *b* | *SE* | *z* | *p* |
| Intercept | -1.39 | 0.15 | -9.48 | <.001 | -1.42 | 0.18 | -8.11 | <.001 | -1.38 | 0.18 | -7.67 | <.001 |
| Age | -0.04 | 0.003 | -15.15 | <.001 | -0.04 | .003 | -11.16 | <.001 | -0.04 | 0.003 | -11.00 | <.001 |
| Wave | -0.02 | 0.002 | -12.94 | <.001 | -0.01 | .005 | -1.89 | 0.06 | -0.01 | 0.006 | -2.09 | .04 |
| Prior Diagnosis | 1.96 | 0.09 | 22.54 | <.001 | 1.40 | 0.28 | 5.07 | <.001 | 1.26 | 0.31 | 4.05 | <.001 |
| Age * Wave |  |  |  |  | <-.001 | <-.001 | -3.14 | .002 | <-.001 | <.001 | -2.02 | .04 |
| Age * Diagnosis |  |  |  |  | 0.01 | 0.01 | 1.60 | .11 | 0.01 | 0.006 | 1.85 | .06 |
| Wave * Diagnosis |  |  |  |  | 0.01 | 0.003 | 2.99 | .003 | 0.02 | 0.01 | 1.81 | .07 |
| Age * Wave * Diagnosis |  |  |  |  |  |  |  |  | <-.001 | <.001 | -0.93 | .35 |
| *Control variables* |  |  |  |  |  |  |  |  |  |  |  |  |
| Male | -0.46 | 0.08 | -5.43 | <.001 | -0.46 | 0.08 | -5.42 | <.001 | -0.46 | 0.08 | -5.42 | <.001 |
| White | -0.08 | 0.09 | -0.90 | .37 | -0.08 | 0.09 | -0.84 | 0.40 | -0.08 | 0.09 | -0.84 | .40 |
| Annual Income >$75k | -0.20 | 0.07 | -3.05 | .002 | -0.20 | 0.07 | -3.05 | .002 | -0.21 | 0.07 | -3.07 | .002 |
| Married | -0.40 | 0.08 | -5.33 | <.001 | -0.41 | 0.08 | -5.43 | <.001 | -0.41 | 0.08 | -5.43 | <.001 |
| Bachelor’s Degree | -0.13 | 0.08 | -1.59 | .11 | -0.13 | 0.08 | -1.61 | .11 | -0.13 | 0.08 | -1.59 | .11 |

Note: Psychological distress was measured with the Patient Health Questionnaire (PHQ-4), and scores ≥ 6 indicated anxiety and depression. Variable titled “Wave” refers to survey wave. Variable titled “Diagnosis” indicates a diagnosis of anxiety or depression prior to 3/10/2022. Addition of the two-way interaction terms significantly improved model fit, χ^2^ = 26.23, *p* < .001. Addition of the three-way interaction term did not improve model fit, χ^2^ = 1.02, *p* = .31.

**Supplemental Table 6**

*Bivariate Correlation Matrix of Outcome and Predictor Variables at Wave 4*

| Variable | Age | | | Psychological distress^a^ | | Diagnosis | | Male | | White | | HH Income  ≥$75,000 | | Married | |
| --- | --- | --- | --- | --- | --- | --- | --- | --- | --- | --- | --- | --- | --- | --- | --- |
| Age |  | — |  |  |  |  |  |  |  |  |  |  |  |  |  |
| Psychological distress^a^ |  | -0.15 | *** | — |  |  |  |  |  |  |  |  |  |  |  |
| Diagnosis |  | -0.08 | *** | 0.24 | *** | — |  |  |  |  |  |  |  |  |  |
| Male |  | 0.12 | *** | -0.10 | *** | -0.16 | *** | — |  |  |  |  |  |  |  |
| White |  | 0.16 | *** | -0.02 |  | 0.07 | *** | 0.04 | *** | — |  |  |  |  |  |
| HH Income  ≥$75,000 |  | -0.01 |  | -0.12 | *** | -0.10 | *** | 0.12 | *** | 0.10 | *** | — |  |  |  |
| Married |  | 0.16 | *** | -0.11 | *** | -0.09 | *** | 0.15 | *** | 0.17 | *** | 0.28 | *** | — |  |
| College |  | 0.01 |  | -0.05 | *** | -0.05 | *** | 0.08 | *** | 0.02 |  | 0.36 | *** | 0.10 | *** |

Note: *** *p* <.001. Correlation results are reported from Wave 4, the first measurement of prior depression or anxiety diagnosis. ^a^Psychological distress was measured with the Patient Health Questionnaire (PHQ-4), and scores ≥ 6 indicated anxiety and depression
